# Response Surface Methodology Optimization of Fermentation Conditions for Rapid and Efficient Accumulation of Macrolactin A by Marine *Bacillus amyloliquefaciens* ESB-2

**DOI:** 10.3390/molecules18010408

**Published:** 2012-12-28

**Authors:** Shan He, Hongqiang Wang, Bin Wu, Hui Zhou, Peng Zhu, Rui Yang, Xiaojun Yan

**Affiliations:** 1 Key Laboratory of Applied Marine Biotechnology, Ningbo University, Ministry of Education, Ningbo 315211, China; E-Mails: heshan@nbu.edu.cn (S.H.); whq.8685136@163.com (H.W.); zhouhui925@yeah.net (H.Z.); zhupeng@nbu.edu.cn (P.Z.); yangrui@nbu.edu.cn (R.Y.); 2 Department of Ocean Science and Engineering, Zhejiang University, Hangzhou 310058, China; E-Mail: wubin@zju.edu.cn

**Keywords:** macrolactin A, fermentation, *Bacillus amyloliquefaciens*, response surfaces methodology, marine antibiotics

## Abstract

In the present work, an antibiotic-producing marine bacterium was isolated from a seawater sample collected from Yuhuan, Zhejiang, China, identified and named as *Bacillus amyloliquefaciens* ESB-2 on the basis of phenotypic characteristics and 16S rRNA gene sequencing. Response surface methodology was applied to optimize the fermentation conditions for rapid and efficient accumulation of macrolactin A, a pharmacologically important marine antibiotic. Eight fermentation conditions were examined for their significance on macrolactin A production using Plackett–Burman factorial design, where peptone, medium volume and temperature significantly improved production rate. Further optimization was carried out using Box-Behnken design of experiments to study the influence of process variables. The optimized fermentation condition for maximum production was peptone 14.8 mg/mL, yeast extract 1 mg/mL, FePO_4_ 0.01 mg/mL, temperature 26.3 °C, initial pH value 6.0, medium volume 72.4%, rotation speed 150 r/min, inoculation 5% and fermented for 2 days. Under the optimized conditions, the concentration of macrolactin A reached 21.63 mg/L, representing a 2.4-fold increase compared to the original standard condition, which was also 17% higher than previous highest report of 18.5 mg/L and three times higher in terms of daily productivity.

## 1. Introduction

During the past one hundred years, natural products research has mainly concentrated on the terrestrial biota, and a lot of structurally unique and highly bioactive compounds were found in terrestrial microorganisms, plants and animals. However, for long-term research and development, discovering new and useful compound from terrestrial resources is becoming much more difficult [1]. Meanwhile, with the development of technology more and more scientists are shifting their attention to the oceans, which host the vast majority of biodiversity [2,3,4]. In recent years, numerous novel and complex compounds were isolated from marine organisms [5,6].

**Figure 1:**
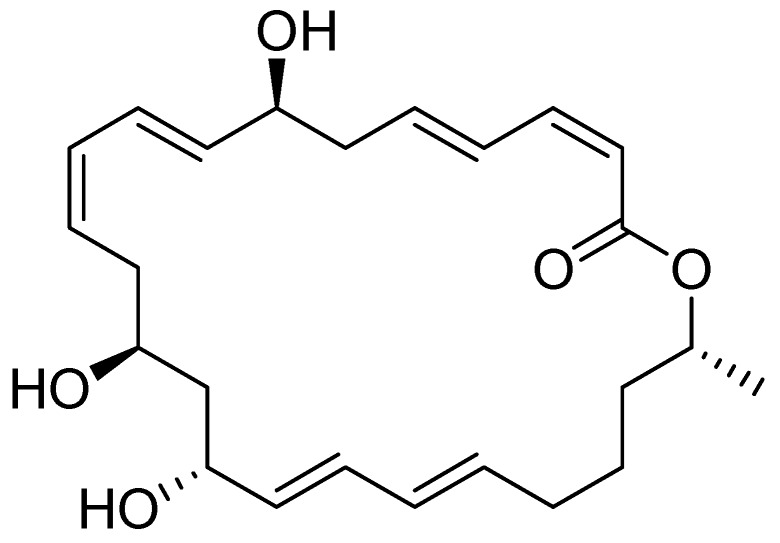
The chemical structure of macrolactin A.

Macrolactins are a group of 24-membered macrolides with potent antibacterial or other activities, most of which are produced as secondary metabolites by marine microorganisms [7]. A total of 19 isolated macrolactins were isolated and characterized [8,9,10,11,12,13,14]. Macrolactin A (Figure 1) was first isolated in 1989 by Gustafson *et al.* from a taxonomically undefined deep-sea marine bacterium. It displays a broad spectrum of biological activity, including potent cytotoxicity on B16-F10 murine melanoma cell and antiviral activity against *Herpes simplex* (types I and II) and HIV virus [7]. Of the 19 members, macrolactin A was considered the most bioactive. However, the extreme scarcity of material has precluded further pharmacological investigation of the compound. Hence, search for better stains and optimization of fermentation conditions to improve macrolactin A production is warranted. 

An appropriate fermentation medium is very important, because medium composition can significantly affect product yield [15]. Response Surface Methodology (RSM) is an efficient strategic experimental tool to obtain the optimum conditions for a multivariable system [16,17,18,19,20,21]. Recently, a marine bacterium was isolated from seawater in Yuhuan (Zhejiang, China) and identified and named as *Bacillus amyloliquefaciens* ESB-2, which was found to rapidly accumulate macrolactins. The aim of the present work was to optimize the culture medium composition for rapid and efficient production of macrolactin A by *Bacillus amyloliquefaciens* ESB-2 using RSM.

## 2. Results and Discussion

### 2.1. Optimization of Culture Conditions by Plackett-Burman Design (PBD)

The importance of the eight variables, namely, peptone (x_1_), yeast extract (x_2_), beer extract (x_3_), glucose (x_4_), FePO_4 _(x_5_), medium volume (x_6_), temperature (x_7_) and initial pH value (x_8_) for macrolactin A production was investigated by PBD. The effects of these components on the response and significant levels are shown in Table 1 and Table 2. 

According to statistical analysis of the data by the Design Expert software Minitab 16.0 [22], peptone (x_1_), medium volume (x_6_) and temperature (x_7_) had confidence levels above 95% (*p* < 0.05) and were considered to influence macrolactin A production significantly. The others had confidence levels below 95% and hence were deemed insignificant. In addition, *R*^2^ = 0.9889 indicated that 98.89% of the variability in the response could be explained by the model.

**Table 1:** The Plackett-Burman design for screening variables in macrolactin A production.

Variable	Code	Low level (−)	High level (+)	Coefficient	*t*-value	*p*-value
Intercept				11.696	46.10	0.000
Peptone (g/L)	x_1_	5	10	1.328	5.17	0.014
Yeast extract (g/L)	x_2_	1	2	−0.525	−2.05	0.133
Beer extract (g/L)	x_3_	0	1	−0.872	−3.05	0.056
Glucose (g/L)	x_4_	0	10	0.592	2.31	0.104
FePO_4_ (g/L)	x_5_	0.01	0.02	−0.157	−0.61	0.584
Medium volume (g/L)	x_6_	40%	60%	3.068	11.95	0.001
Temperature (°C)	x_7_	30	35	−2.272	−8.85	0.003
Initial pH value	x_8_	6	7	−0.148	−0.57	0.606

R^2^ = 98.89%; R^2^_adj_ = 95.93%.

**Table 2:** The Plackett–Burman design along withmacrolatin A production as response.

Run	Variable Level								Macrolactin A (mg/L)
	x_1_	x_2_	x_3_	x_4_	x_5_	x_6_	x_7_	x_8_	
1	−1	−1	−1	1	1	1	−1	1	17.81
2	−1	1	1	1	−1	1	1	−1	10.47
3	1	−1	1	−1	−1	−1	1	1	7.35
4	−1	1	−1	−1	−1	1	1	1	11.13
5	1	−1	1	1	−1	1	−1	−1	19.29
6	−1	1	1	−1	1	−1	−1	−1	7.79
7	1	1	1	-1	1	1	−1	1	16.03
8	1	1	−1	1	−1	−1	−1	1	12.42
9	1	−1	−1	−1	1	1	1	−1	13.87
10	−1	−1	−1	−1	−1	−1	−1	−1	10.46
11	−1	−1	1	1	1	−1	1	1	4.56
12	1	1	−1	1	1	−1	1	−1	9.19

PBD results also indicated that the effect of peptone (x_1_) and medium volume (x_6_) were positive, while temperature (x_7_) exhibited a negative effect on macrolactin A production. Therefore, these three variables were selected for further optimization.

### 2.2. Optimization by Response Surface Methodology

RSM using Box-Behnken design (BBD) was employed to determine the optimal levels of the three selected variables. The respective levels with the coded levels for the factors are listed in Table 3. The concentrations of the other factors were set at zero level as shown in Table 1. Experimental design and results are shown in Table 4. 

**Table 3:** The level of variables for the Box-Behnken design.

Variables	Code	Level		
		−1	0	1
Peptone (g/L)	x_1_	5	10	15
Medium volume (mL)	x_6_	40%	60%	80%
Temperature (°C)	x_7_	25	30	35

**Table 4:** Box-Behnken design along with macrolatin A production as response.

Run	Variable Level			Macrolactin A (mg/L)
	x_1_	x_6_	x_7_	
1	0	0	0	19.08
2	0	−1	1	6.35
3	0	1	1	6.69
4	−1	1	0	11.70
5	1	0	1	12.56
6	0	−1	−1	6.70
7	1	0	−1	20.42
8	−1	−1	0	9.17
9	1	−1	0	8.40
10	−1	0	1	7.83
11	0	0	0	19.19
12	−1	0	−1	14.57
13	1	1	0	18.21
14	0	0	0	19.66
15	0	1	−1	19.26

The relationships between macrolactin A production (*Y*) and the tested variables were obtained by application of RSM. By employing multiple regression analysis on the experimental data, the response variable (*Y*) and the tested variables can be related by the following second-order polynomial equation:

*Y *= 19.31 + 2.04*x_1_ + 3.155*x_6_ − 3.44*x − 1.6725*x_1_*x_1_ − 5.7675*x_6_*x_6_ − 3.7925*x_7_*x_7_ + 1.82* x_1_*x_6_ − 3.055*x_6_*x_7 _

where *Y* was the predicted macrolactin A production, x_1_was peptone, x_6_ was medium volume, and x_7_ was temperature.

The analysis of variance (ANOVA) data for the selected quadratic polynomial model is listed in Table 5. The high model *F*-value (67.52) and low *p*-value (<0.05) implied the model was highly significant. The fitness of the model could be examined by the coefficient of determination R^2^ [23], which was calculated to be 0.9918, indicating that 99.18% of the sample variation was attributed to the variables and only less than 1% of the total variance could not be explained by the model. A regression model, having an R^2^-value higher than 0.9, was considered as having a very high correlation. Therefore, the present R^2^-value reflected a very good fit between the observed and predicted responses, and implied that the model is reliable for macrolactin A production in the present study. The adjusted determination coefficient (R^2^_adj_ = 97.72%) was also able to confirm the significance of the model.

**Table 5:** Analysis of variance (ANOVA) for the second-order polynomial model.

Source	SS	DF	MS	*F*-value	*p*-value
Model	426.621	9	47.402	67.52	0.000
Residual	3.510	5	0.702		
Lack of Fit	3.320	3	1.107	11.66	0.080
Pure Error	0.190	2	0.095		
Cor Total	430.131	14			

R^2^ = 99.18%; R^2^_adj_ = 97.72%.

The coefficient estimates of model equation, along with the corresponding *p*-values, are presented in Table 6. The *p*-value was employed as a tool to check the significance of each coefficient, which also indicated the interactions between the variables [24]. The smaller the *p*-value, the more significant the corresponding coefficient was. It was observed from Table 6 that all regression coefficients were highly significant with *p*-values less than 0.05 except for the cross-product coefficient of peptone (x_1_) and temperature (x_7_).

**Table 6:** Regression results of the Box-Behnken design.

Variables	Parameter estimate	Standard error	*t*-value	*p*-value
Intercept	19.3100	0.4837	39.919	0.000
x_1_	2.0400	0.2962	6.887	0.001
x_6_	3.1550	0.2962	10.651	0.000
x_7_	−3.4400	0.2962	−11.613	0.000
x_1_*x_1_	−1.6725	0.4360	−3.836	0.012
x_6_*x_6_	−5.7675	0.4360	−13.227	0.000
x_7_*x_7_	−3.7925	0.4360	−8.698	0.000
x_1_*x_6_	1.8200	0.4189	4.334	0.007
x_1_*x_7_	−0.2800	0.4189	−0.668	0.534
x_6_*x_7_	−3.0550	0.4189	−7.292	0.001

**Figure 2:**
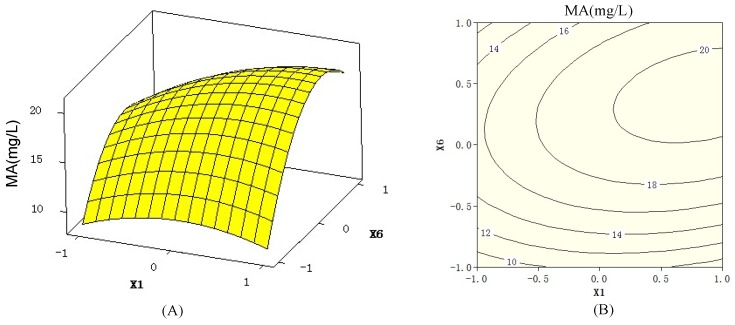
3D response surfaces (A) and 2D contour plots (B) showing the effects of peptone (x_1_), medium volume (x_6_), and their mutual interaction on macrolactin A (MA) production, when temperature (x_7_) was maintained at 30 °C.

**Figure 3:**
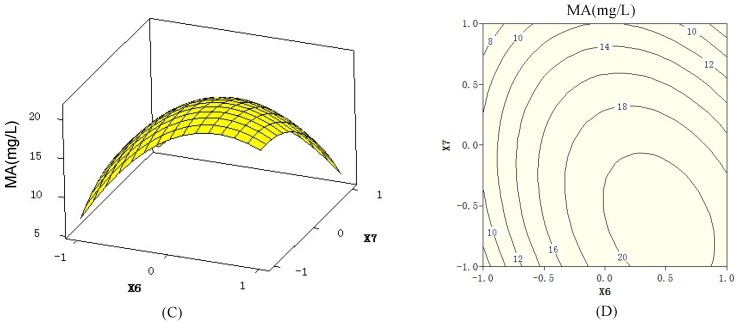
3D response surfaces (C) and 2D contour plots (D) showing the effects of medium volume (x_6_), temperature (x_7_),and their mutual interaction on macrolactin A (MA) production, when peptone (x_1_) concentration was at 10 g/L.

The 3D response surfaces and 2D contour plots (Figure 2 and Figure 3) generated by Minitab 16.0 are the graphical representations of the regression equation. They can visualize the relationship between the response and each variable, and the interactions between two tested variables. The 3D response surfaces and their respective 2D contours can also locate the optimum ranges of the variables for the maximum of the response. The maximum predicted response was indicated by the surface confined in the smallest ellipse in the contour diagram. An elliptical contour would be obtained when there was an obvious interaction between two independent variables [25]. 

### 2.3. Validation of the Optimized Condition

On the basis of medium optimization, the quadratic model predicted that the maximum production of macrolactin A was 22.42 mg/L, when the x_1_ code level was 0.95, x_6_ code level was 0.62 and that of x_7_ was −0.74, which were peptone 14.8 mg/L, medium volume 72.4%, temperature 26.3 °C, respectively. To verify the predicted results, validation experiments were performed in triplicate. Under the optimized condition, the observed experimental average concentration of macrolactin A was 21.63 mg/L, suggesting that experimental and predicted values (22.42 mg/L) were in good agreement, which was also 17% higher than previous highest report of 18.5 mg/L [26]. Since ESB-2 could rapidly accumulate macrolactin A in only 2 days, while previous reports required more than 5 days, our result is at least three times higher in terms of daily productivity than previous reports. It is worth noting that the increase of macrolactin A production resulted in the decrease of other macrolactins especially macrolactin B, a glucoside of macrolactin A. The results revealed that glycosylation of macrolactin A was inhibited under the optimized fermentation condition.

## 3. Experimental

### 3.1. Microorganism

A macrolactins-producing marine bacterium was isolated from a seawater sample collected in Yuhuan, Zhejiang, China. The 16S rRNA gene sequencing result showed that this strain had 97% homology with *Bacillus amyloliquefaciens* reported in the Genbank (No. JX966407). According to its morphological and physicochemical characteristics, the strain was identified and named as *Bacillus amyloliquefaciens* ESB-2 [27,28].

### 3.2. Culture Conditions

The bacterium was grown in 2216E medium (5 g/L peptone, 1 g/L yeast extract, 0.1 g/L FePO_4_, 1 L seawater). Fermentation was performed in two stages: seed growth and macrolactin A production. For the seed growth stage, mycelium from a plate culture was inoculated into 100 mL of seed medium with shaking at 150 rpm at 30 °C for 24 h. Then, 5% (v/v) seed cultures were inoculated into the fermentation medium. The strain was incubated at 30 °C with 150 rpm for 2 days.

### 3.3. Analytical Method

The fermentation broth was extracted with EtOAc for three times. The extracts were evaporated and dissolved in 1 mL methanol. After centrifuging (12,000 rpm, 10 min), the supernatants were analyzed by a Waters Alliance 2695 HPLC system, equipped with a quaternary solvent deliver system, an autosampler, and a 2996 diode array detector (Waters, Milford, MA, USA). A reverse-phase Waters XBridge C18 column (150 mm × 4.6 mm ID, 5 μm, Waters) at 25 °C was applied for all analyses. Methanol–water was used as the mobile phase in gradient elution mode (methanol: 0–60 min, 10–90%). The effluent was monitored at 280 nm and flow rate was 1.0 mL/min. Then authentic sample of macrolactin A with purity higher than 95% was isolated and purified from *Bacillus amyloliquefaciens* by high-speed counter-current chromatography as described elsewhere [29].

### 3.4. Experimental Design and Data

Eight fermentation conditions were examined for their significance on macrolactin A production using PBD [30,31,32]. Further optimization was carried out using BBD experiments and RSM to study the influence of process variables on macrolactin A production [33,34,35].

## 4. Conclusions

This study proved that statistical experimental designs offer an efficient and feasible approach for macrolactin A fermentation medium optimization. A maximum macrolactin A production of 21.63 mg/L was achieved with the following optimized factors: peptone 14.8 mg/mL, yeast extract 1 mg/mL, FePO_4_ 0.01 mg/mL, temperature 26.3 °C, initial pH value 6.0, medium volume 72.4% and fermented for 2 days. Under the optimized conditions, the concentration of macrolactin A reached 21.63 mg/L. Validation experiments were also carried out to verify the accuracy of the model, and results showed that the predicted value agreed well with the experimental values. Comparing to 9.17 mg/L in original standard condition, 2.4-fold increase had been obtained. This medium also resulted in 17% higher than previous report and three times higher in terms of daily productivity. The present study provides a basis for further study on large scale fermentation for macrolactin A production.
